# Premature ovarian insufficiency and the risk of breast cancer

**DOI:** 10.1093/hropen/hoaf017

**Published:** 2025-04-10

**Authors:** Herjan J T Coelingh Bennink, Jan F M Egberts, Frank Z Stanczyk

**Affiliations:** Pandora Endocrine Innovation, Bergen, The Netherlands; Terminal 4 Communications, Hilversum, The Netherlands; Department of Obstetrics and Gynecology, University of Southern CA, Keck School of Medicine, Los Angeles, CA, USA

Sir,

Recently, *Human Reproduction Open* published an update of the ESHRE, American Society for Reproductive Medicine (ASRM), Centre of Research Excellence in Women’s Health in Reproductive Life (CREWHIRL), and International Menopause Society (IMS) evidence-based guideline on premature ovarian insufficiency (POI) (Panay *et al.*, 2024a), in parallel with similar publications in *Climacteric* and *Fertility & Sterility* (Panay *et al.*, 2024b; Panay *et al.*, 2025). However, this extensive and very detailed guideline does not address the decreased risk of breast cancer (BCa) in women with POI (ESHRE, 2024). Among all the negative health implications of POI, the significantly lower risk of BCa is a benefit, and treatment of POI should not interfere with this important favorable effect of POI.

In a recent article, we presented the outcome of an extensive literature search regarding the relationship between the number of menstrual cycles (MCs) and the risk to develop BCa (Coelingh Bennink *et al.*, 2023). With average menarche at 11 years, average menopause at 51 years, and a mean MC duration of 28 days, a woman experiences 13 MCs per year and 520 MCs during 40 years of menstrual cycling (2.5% BCa risk per year). The absolute lifetime risk of developing BCa in women in the USA has increased from 1 in 11 (9.1%) during 1975–1977 to 1 in 8 (13.0%) during 2017–2019 (NBCC, 2024), and women in the UK born after 1960 even have a risk of 1 in 7 (14.3%) (Cancer Research UK, 2025). Physiological variations in the frequency of MCs (menarche, menopause, pregnancies, lactation) and interference with MCs through genetic variations, pathological conditions (including POI), and pharmaceutical interventions reveal a strong direct relationship between the BCa risk and the lifetime number of MCs (Clavel-Chapelon, 2002; Collaborative Group on Hormonal Factors in Breast Cancer, 2012).

As stated by ESHRE (2024) POI is a condition defined by loss of ovarian activity before the age of 40 years, characterized by amenorrhea or irregular MCs with elevated gonadotropins and low estradiol. In a study among 12 134 women whose menopause occurred before the age of 40 years, a significantly increased total all-cause mortality was observed compared with the reference group, whose menopause occurred at age 50–54 years [hazard ratio (HR) 1.40 (95% CI 1.15–1.17)]. However, the incidence of mortality due to BCa was much lower than in the reference group [HR 0.55 (95% CI 0.17–1.82)] (Ossewaarde *et al.*, 2005). In a population-based BCa-screening cohort of 10 591 women, early menopause occurring at ages below 45 or at 45–49 years showed a protective effect on the risk of BCa [HR 0.66 (95% CI 0.43–0.91) and HR 0.67 (95% CI 0.48–0.94)], respectively, as compared with menopause occurring at age 55 or older (Monninkhof *et al.*, 1999). Women who became menopausal below 44 years of age have a 34% lower risk of BCa than women aged 55 years. The annual hazard rate in relation to the BCa incidence decreases by 2.6% in women who had an early menopause (Monninkhof *et al.*, 1999), which complies with the 2.5% BCa risk per year. In Chinese women, POI is associated with an increased risk of all-cause and cancer mortality [HR 1.29 (95% CI 1.08–1.54) and HR 1.38 (95% CI 1.05–1.81)], but POI is inversely associated with the incidence of BCa [odds ratio 0.59 (95% CI 0.38–0.91)] (Wu *et al.*, 2014). In women who underwent prophylactic oophorectomy due to *BRCA* mutations, a significant reduction in BCa risk was observed versus the control in the total sample [HR 0.53 (95% CI 0.33–0.84)], becoming more prominent in the subgroup followed for ≥10 years [HR 0.33 (95% CI 0.12–0.91)] (Rebbeck *et al.*, 1999).

A key question is whether the decreased risk of BCa with less MCs is related to the lower exposure to estrogens or to progesterone. Molecular data show that progesterone (P4) may stimulate mutative factors in normal breast epithelium during the luteal phase of the MC, such as the paracrine factors WNT family member 4 (WNT4) and receptor activator of nuclear factor-κB ligand (RANKL) (Coelingh Bennink *et al.*, 2023). Progesterone also upregulates the expression of the DNA mutator enzyme apolipoprotein B mRNA editing catalytic polypeptide-like 3B (APOBEC3B) during this phase of the MC. These molecular effects of P4 likely increase the accumulation of mutations in long-lived mammary stem and progenitor cells. Estrogens, testosterone, and most non-toxic environmental factors that have been related to the risk of BCa have only mild proliferative effects on normal breast epithelium and do not induce mutations but may stimulate the growth of already existing estrogen receptor-positive (ER+)/progesterone receptor-positive (PR+) BCa, which is often misinterpreted as causing BCa. Since it takes at least a decade from a stem cell mutation in the breast until the tumor has grown to a size enabling the diagnosis of a BCa (Santen *et al.*, 2012), BCas diagnosed during the first decade of hormonal contraception or hormonal endometriosis treatment are most likely due to mutations that occurred during earlier spontaneous MCs in those women. Also, BCas diagnosed during the first decade after menopause with or without hormone treatment are due to earlier MCs before menopause.

Women with POI need estrogens. We propose to use estrogen-only treatment, which has been shown in a Women’s Health Initiative (WHI) study to protect against the occurrence of and mortality due to BCa (Anderson *et al.*, 2012; Manson *et al.*, 2017). By adding progesterone or progestins to this substitution treatment, the risk of BCa will most likely return to the high risk of normally cycling women. Therefore, we propose to avoid the use of progestogens and treat POI patients with estrogens only, preferably the orally bioavailable and cardiovascular safe fetal estrogen estetrol (E4) (Coelingh Bennink *et al.*, 2016; Gemzell-Danielsson *et al.*, 2022), monitor vaginal bleeding and endometrial growth regularly, and only prescribe progestin withdrawal when needed, preferably using dydrogesterone for 2-week periods to shed the endometrium. This treatment strategy is expected to preserve the lower BCa risk of POI.
